# On the Influence of Inhomogeneous Interphase Layers on Instabilities in Hyperelastic Composites

**DOI:** 10.3390/ma12050763

**Published:** 2019-03-06

**Authors:** Nitesh Arora, Adi Batan, Jian Li, Viacheslav Slesarenko, Stephan Rudykh

**Affiliations:** 1 Department of Mechanical Engineering, University of Wisconsin Madison, Madison, WI 53706, USA; narora7@WISC.EDU; 2 Department of Aerospace Engineering, Technion—Israel Institute of Technology, Haifa 32000, Israel; adibatan@technion.ac.il (A.B.); jianli@campus.technion.ac.il (J.L.); slslesarenko@technion.ac.il (V.S.)

**Keywords:** 3D printing, inhomogeneous interphase, instability, fiber composites, microscopic instability

## Abstract

Polymer-based three-dimensional (3D) printing—such as the UV-assisted layer-by-layer polymerization technique—enables fabrication of deformable microstructured materials with pre-designed properties. However, the properties of such materials require careful characterization. Thus, for example, in the polymerization process, a new interphase zone is formed at the boundary between two constituents. This article presents a study of the interphasial transition zone effect on the elastic instability phenomenon in hyperelastic layered composites. In this study, three different types of the shear modulus distribution through the thickness of the interphasial layer were considered. Numerical Bloch-Floquet analysis was employed, superimposed on finite deformations to detect the onset of instabilities and the associated critical wavelength. Significant changes in the buckling behavior of the composites were observed because of the existence of the interphasial inhomogeneous layers. Interphase properties influence the onset of instabilities and the buckling patterns. Numerical simulations showed that interlayer inhomogeneity may result in higher stability of composites with respect to classical layup constructions of identical shear stiffness. Moreover, we found that the critical wavelength of the buckling mode can be regulated by the inhomogeneous interphase properties. Finally, a qualitative illustration of the effect is presented for 3D-printed deformable composites with varying thickness of the stiff phase.

## 1. Introduction

Composite materials are deeply integrated in the modern life, due to their excellent mechanical and functional properties, which until recently were unachievable. Composites containing two or more constituents can be tailored to meet specific requirements by design of their geometry and smart selection of constituent materials. One of the most challenging problems in composite science is associated with the prediction of their failure. While delamination was historically considered to be the main failure mode, the phenomenon of local buckling or loss of stability attracted significant attention recently. In contrast with delamination, when composites lose the integrity catastrophically, the loss of stability can be considered as reversible microstructure transformation mode; once the external loads are removed, the initial undeformed state is restored thanks to the stored elastic energy. This phenomenon of instability-induced microstructure transformation has been employed to design materials with the switchable properties and functions [1,2,3,4,5,6,7,8].

The pioneering works of Rosen [9] and Hill and Hutchinson [10] laid the basis for the theoretical understanding of the elastic instability phenomenon. The development of analytical tools as well as numerical methods contributed to the increasing number of studies devoted to the mechanical instabilities in materials and structures. Stability of layered hyperelastic composites under compressive loads were studied by Triantafyllidis and Maker [11] and, more recently, Nestorović and Triantafyllidis [12], who predicted the onsets of instabilities in hyperelastic layered media at the microscopic and macroscopic length scales under plane-strain conditions. Merodio and Ogden [13,14,15] showed that the instabilities are possible even under tensile loadings for incompressible and compressible material models satisfying certain conditions. The onset of macroscopic instabilities in fibrous and layered composites were analytically studied with the help of loss of ellipticity analysis [16,17], when the required tensor of effective elastic moduli is obtained based on phenomenological models, or by means of micromechanics based homogenization approaches [18,19,20]. More sophisticated techniques, such as the Bloch-Floquet analysis, were required to study the microscopic instabilities, which develop at the length scales comparable with the dimensions of the composite microstructure [21]. This approach was successfully applied for the periodic composites under plane-strain deformation [22], for two-dimensional layered media subject to combination of shear and compressive deformation [12], as well as for fiber composites subject to the compression in fully three-dimensional (3D) settings [23,24].

At the same time, development of new composite manufacturing methods, especially appearance of multimaterial three-dimensional printing, opens the possibility to validate theoretical and numerical findings [1,25,26]. For instance, macroscopic and microscopic buckling modes were experimentally observed in soft layered composites, manufactured with the help of multi-dimensional 3D-printing [27]. Moreover, the experimentally observed critical strains and corresponding buckling patterns were in good agreement with the theoretical predictions. A significant number of the experimental studies on the instability analysis of the different soft composite structures produced by 3D-printing were published in recent years [28]. However, accurate experimental studies involving 3D-printed materials pose a challenging problem due to uncertainness of the multimaterial 3D-printing technique [29]. The relatively narrow range of the materials available for 3D printing, variability of their properties due to changes in the environmental conditions [30] and anisotropy of the printing process [31,32] might introduce inaccuracies in the experimental results. While the development of the multi-material 3D-printing undeniably drastically extends the possible application of additive manufacturing, it also introduces new issues to be addressed. Due to the resolution limitation of the technique, perfect transition between two different materials is impossible, and a mixing zone is produced at the boundary between two constituents. How the existence of such interphasial zone affects the mechanical properties of 3D-printed composites is an issue becomes important with regard to the stability analysis. Recently, it was analytically shown that the existence of the interphasial layers in the layered composites significantly affects their stability [33]. Moreover, Gao and Li [34] numerically revealed the formation of hierarchical buckling patterns in the composites containing additional layer. However, the mentioned study [34] treats the transition (regulator) layer as another homogeneous material, while in realistic composites, especially produced by 3D-printing, this interphasial layer is a graded material with properties changing through the thickness. In this study, we analyzed the stability of the hyperelastic layered composites containing nonhomogeneous interphasial layers, appearing at the boundary of soft matrix and stiffer layers. We employed the Bloch-wave approach to obtain the information on the instability patterns and associated critical strains in dilute and non-dilute layered composites with different geometrical parameters and material properties. Finally, some qualitative comparison with the experimentally observed instability modes in 3D printed layered composites is discussed.

## 2. Numerical Simulations

**Figure 1:**
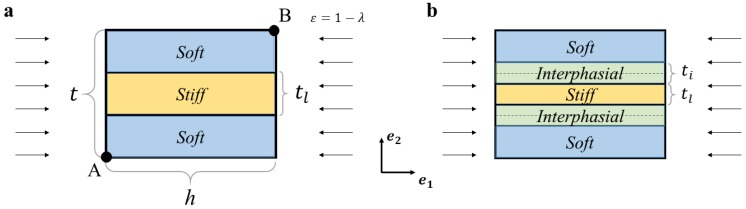
Unit cells of “ideal” layered composite (**a**) and “non-ideal” composite with interphasial layers (**b**).

To analyze the mechanics of the layered composite with interphasial layers bonding two purely homogenous hyperelastic constituent materials, we utilized the finite element method with the help of COMSOL Multiphysics (v. 5.2, COMSOL AB, Stockholm, Sweden). Representative volume element (RVE) with dimensions t=a, h=0.05a was used in the calculations, as shown in Figure 1. A mesh sensitivity analysis was performed, and the RVE containing 2498 quadrilateral elements with quadratic shape functions was used.

Figure 1 shows the unit cell of an “ideal” composite without any interphasial layers (a), and the realistic composite, which contains the transition zone between two main constituents (b). In the ideal composite, the thickness of the stiff layer is $t_{l}=v_{l}t$, where $v_{l}$ is the volume fraction of the stiffer homogenous layer, and t is the period of the unit cell. We assumed that the interphasial layers, generated from the 3D-printing process (see Experimental section), contain equal amounts of the stiffer layer and soft matrix materials; the thicknesses of the pure stiff and the interphasial layers in this case are equal to $t_{l}=\left(1-f\right)v_{l}t$ and $t_{i}=fv_{l}t,$ respectively. Here, 0≤f≤1 is the relative thickness of the interphasial layer. 

**Figure 2:**
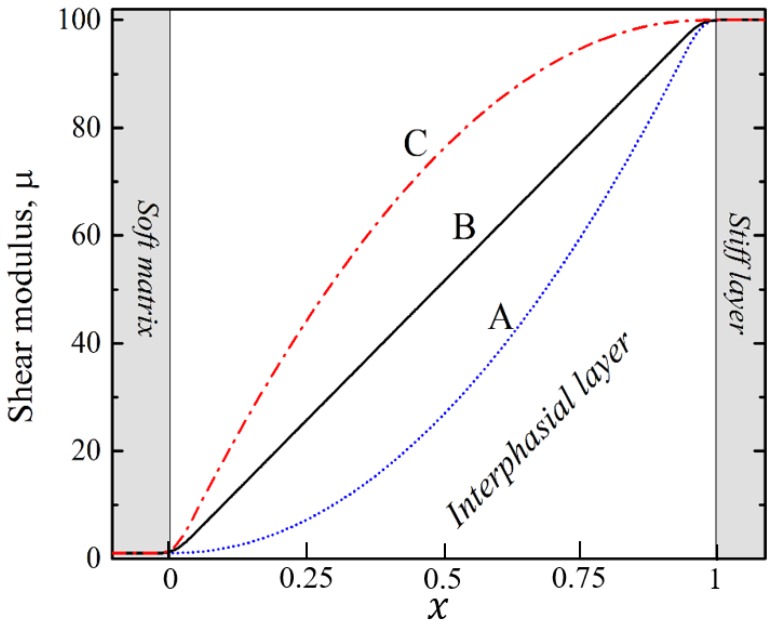
Different types of shear modulus variation in the interphasial layer.

Since the interphasial layer is inhomogeneous, we need to define the variation of the local elastic modulus through the thickness of the interface. In this study, we considered three different types of shear moduli distributions through thickness, denoted as A, B and C on Figure 2. Shear modulus in the interphasial zone $\mu_{i}$ at position x (see Figure 2) is defined as:

(1) $$\mu_{i}\left(x\right)=\left(2\left(\mu_{l}-\mu_{m}\right)-4\mu_{h}\right)x^{2}+\left(4\mu_{h}-\left(\mu_{l}-\mu_{m}\right)\right)x+\;\mu_{m},$$

where $\mu_{m}$ and $\mu_{l}$ denote the initial shear modulus of soft matrix and stiff layer, respectively. In expression (1), x varies from zero to one (see Figure 2), $\mu_{h}=c\left(\mu_{l}-\mu_{m}\right)$ where c=0.25, 0.5 and 0.75 for distributions A, B and C, respectively. Note, that for distribution B, the elastic modulus linearly increases between the values of the initial shear modulus of the soft matrix $\mu_{m}$ and stiffer layer $\mu_{l}$. The distribution A corresponds to the case, when the average elastic modulus of the interphasial layer was lower than $(\mu_{l}+\mu_{m})/2$, as opposed to the distribution C, when the average shear modulus of the interphasial layer exceeded the value of $(\mu_{l}+\mu_{m})/2$. All constituents, including the interphasial layers, were considered as nearly incompressible hyperelastic materials with the neo-Hookean strain-energy function, integrated in COMSOL as:

(2) $$W=\frac{1}{2}\mu \left(I_{1}\left(C\right)-3\right)-\mu \ln\left(J\right)+\frac{1}{2}\Lambda \ln{\left(J\right)}^{2}$$

where Λ is the first Lame constant, $C=F^{T}F$ is the right Cauchy-Green tensor and J=det(F) is the determinant of the deformation gradient F. To maintain the nearly incompressible behavior of the constituents, we set a high ratio between the shear modulus and the first Lame constant; in particular Λ=1000μ was used in our simulations.

In order to analyze the stability of the considered layered composites, we employed the Bloch-Floquet analysis [21] superimposed on finite deformations. The procedure for identifying the onset of instabilities and associated wavenumbers was performed in two steps. First, the unit cell underwent static finite deformation, defined by means of the displacement periodic boundary conditions:

(3) $$\{\begin{array}{c} {u_{1}|}_{right}={u_{1}|}_{left}-\varepsilon h\\ {u_{2}|}_{right}={u_{2}|}_{left} \end{array}\;\;\;\;\;\;\;\{\begin{array}{c} \begin{array}{c} {u_{1}|}_{top}={u_{1}|}_{bottom}\\ {u_{2}|}_{top}={u_{2}|}_{bottom}+{u_{2}|}_{B} \end{array}\\ {u_{1}|}_{A}={u_{2}|}_{A}=0 \end{array}$$

Here, the indexes *left, right, top* and *bottom* denote the edges of the unit cell (see Figure 1); A and B correspond to the nodes located at the corners. The stiffness matrix, obtained during the solution of the problem (2), is stored for further solution of the eigenvalue problem on the next step, when the Bloch-wave conditions are superimposed on the deformed state by using the boundary conditions:

(4) $$\{\begin{array}{c} {u_{1}|}_{right}={u_{1}|}_{left}e^{ikh\cdot 2\pi }\\ {u_{2}|}_{right}={u_{2}|}_{left}e^{ikh\cdot 2\pi } \end{array}\;\;\;\;\;\;\;\;\;\;\;\{\begin{array}{c} {u_{1}|}_{top}={u_{1}|}_{bottom}\\ {u_{2}|}_{top}={u_{2}|}_{bottom} \end{array}$$

Here, $\tilde{k}$ is normalized wavenumber corresponding to the Bloch wave vector. We swept the values of $\tilde{k}$ in the range from 0 to 10 with a step of 0.05, solving the corresponding eigenvalue problem, until the lowest eigenvalue became zero. If for the considered range of $\tilde{k}$ values only positive eigenvalues appear, the instability was not detected, and the composite remained stable for given ε. In this case, we increased the compressive strain and repeated the procedure, described above, for an increased ε, until the zero eigenvalue for non-zero $\tilde{k}$ was found. The strain ε and wavenumber $\tilde{k}$, for which the first zero eigenvalue was observed, corresponded to the buckling strain $\varepsilon^{cr}$ and critical wavenumber ${\tilde{k}}^{cr}$, defining the buckling shape. Recall that the special case of ${\tilde{k}}^{cr}\to 0$ corresponds to the macroscopic instability (long-wave mode); otherwise, the composite undergoes a microscopic loss of stability, developing finite size wavy shapes upon achieving the critical level of deformation [19,20]. 

**Figure 3:**
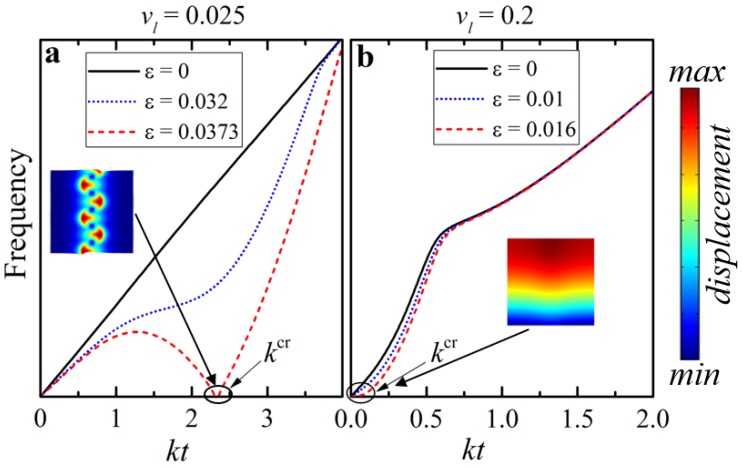
The evolution of the dispersion curves of the lowest eigenfrequency during compression of ideal layered composite with $v_{l}=0.025$ (dilute)—(**a**) and $v_{l}=0.2$ (non-dilute)—(**b**). The elastic modulus contrast is $\mu_{l}/\mu_{m}=100$.

Figure 3 illustrates the described numerical procedure, showing a typical evolution of the dispersion curves with applied deformation for dilute composite with $v_{l}=0.025$ (a) and non-dilute composite with $v_{l}=0.2$ (b). Recall that there is an intrinsic connection between the shear wave propagation and elastic instabilities in periodic composites [24]. The continuous black curve in Figure 3a,b describes the dispersion relation in the undeformed state (ε=0); the curves intersect with the x-axis only at k=0, which corresponds to trivial rigid body motion. An increase in the applied compressive strain leads to a gradual change of the dispersion curves. Figure 3 illustrates these changes for the applied compressive strains ε=0.032 (a) and ε=0.01 (b) (dotted blue curves). Finally, for the strains ε=0.0373 (a) and ε=0.016 (b), we observed that zero eigenvalue appeared at the finite wavenumber (dashed red curve). Therefore, the corresponding composites, being deformed up to these critical strains lost their stability. Note, that for the dilute composite ($v_{l}=0.025$) $k^{cr}t=$ 2.3, which corresponds to the microscopic loss of the stability, while for the non-dilute composite ($v_{l}=0.2)$ $k^{cr}\approx 0$, which corresponds to the macroscopic instability. Buckling modes of the composites with the corresponding critical parameters are shown in Figure 3 for the microscopic (a) and macroscopic (b) cases. Here and thereafter, we use the term “dilute” to refer to the composite undergoing microscopic loss of stability, as opposite to a “non-dilute” composite, which experiences macroscopic instability with formation of long-wave buckling shape. 

## 3. Results

**Figure 4:**
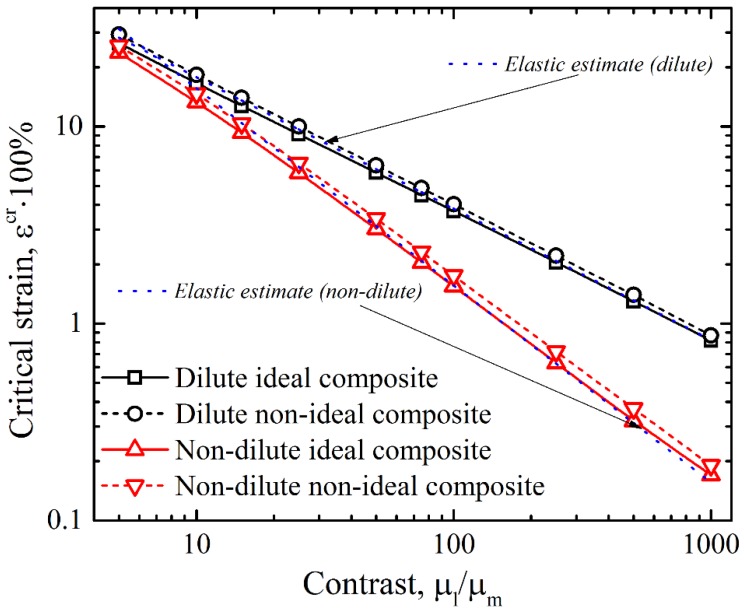
The dependencies of critical strain on the contrast in ideal layered composites with $v_{l}=0.025$ (continuous black) and $v_{l}=0.2$ (continuous red) and non-ideal composites with $v_{l}=0.025$ and f=0.5 (dashed black) and $v_{l}=0.2$ and f=0.5 (dashed red). Dotted blue curves correspond to the elastic estimates [1,27].

Before considering non-ideal composites with interphasial layers, let us firstly make some remarks on the instability in ideal hyperelastic composites without interphases. Figure 4 shows the dependence of the onset of instability on the shear modulus contrast in hyperelastic layered composites under the incompressibility assumption of all constituent materials (Poisson’s ratios of layers and matrix are $p_{l}=p_{m}=0.5$, respectively).

Solid black and red lines correspond to the dilute ($v_{l}=0.025$) and non-dilute ideal composites ($v_{l}=0.2$), respectively. It is clear that in the logarithmic scale this dependence looks almost linear regardless of the instability type that the composite undergoes. Parnes and Chiskis [35] derived the estimation for the onset of instability, occurring in dilute layered composites with linear elastic constituents. Under the assumption of incompressibility of both phases in plane strain conditions, the critical strain can be estimated as:

(5) $$\log(\varepsilon^{cr})\approx -\frac{2}{3}\left[\log\left(\frac{\mu_{l}}{\mu_{m}}\right)+\log\left(\frac{4}{3}\right)\right]$$

This function is shown in Figure 4 with the dotted blue curve. Remarkably, even for high critical strains $\varepsilon^{cr}>20\%$, expression (5), derived for the elastic case, provided very accurate results with negligible variation from the exact value of the critical strain in the dilute layered composite developing microscopic instability mode. Another estimation for the onset of instability in the non-dilute composites, with elastic constituents originally obtained by Rosen [1], is provided in [35]. Under plane strain conditions, this estimation takes the form:

(6) $$\log\left(\varepsilon^{cr}\right)\approx -\log\left(\frac{\mu_{l}}{\mu_{m}}\right)+\log\left(\frac{1}{4v_{l}\left(1-v_{l}\right)}\right).$$

As one may see from Figure 4, where expression (6) is shown by the dotted blue curve, the difference between the exact and estimated values of the critical strain is relatively small for composites with high contrast between elastic moduli; however, it increases with a decrease in the contrast. These observations allowed us to conclude that the critical buckling strain in hyperelastic neo-Hookean composites can be accurately estimated by these expressions, initially derived for the layered composites with linear elastic constituents for dilute as well as for non-dilute cases. However, we note that this good agreement may be due to the fact that the buckling develops at relatively small strains, where the linear model can approximate the nonlinear behavior. 

While the continuous curves in Figure 4 represent the ideal composites, the dashed black and red curves correspond to the composites with the same $\mu_{l}/\mu_{m}$ and $v_{l}$, which contain the interphasial layers with f=0.5 and linear variation of the shear modulus through their thickness (curve B on Figure 2). We can see that the critical strain in the composites with interphasial layers exceeds the critical strain in their “ideal” counterparts. It is worth mentioning that the dashed lines corresponding to non-ideal cases are almost parallel to the solid curves, representing ideal composites, and the difference between the critical strains (in log scale) remains virtually the same regardless of the elastic modulus contrast. 

**Figure 5:**
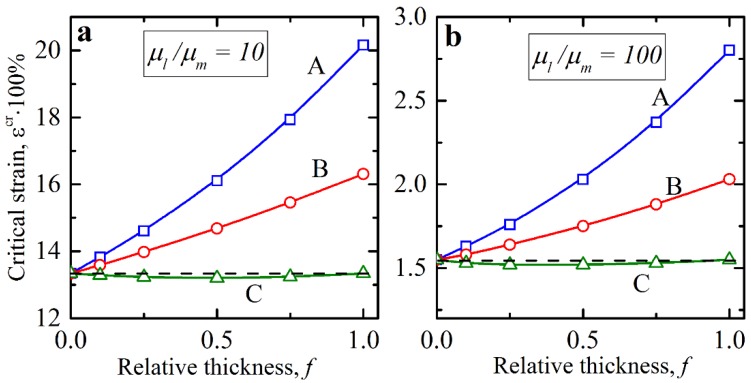
Critical strains in the non-dilute composites with interphasial layers. The shear modulus contrast $\mu_{l}/\mu_{m}$ is 10 (**a**) and 100 (**b**) and the volume fraction $v_{l}=0.2$. Horizontal dashed black line corresponds to the case of ideal composites.

Thereby, the existence of the interphasial layers might make the layered composite more stable; however, thus far, we can state this only for linear variation of shear modulus through the thickness. At the same time, the variation of the shear modulus in the interphasial layers through their thickness might be highly nonlinear, for instance, due to the complexity of the mixing and curing processes at the interphase between the different materials. Figure 5 shows the dependencies of the critical strain $\varepsilon^{cr}$ on the relative thickness of interphasial layers in the non-dilute composite ($v_{l}=0.2)$ for different variations of the shear modulus inside the transition zone. It can be seen, that for distributions A and B, the composites with the interphasial layer were more stable than the ideal composite. Note that composites with linear distribution of shear modulus in interphasial layer (B) had the same effective macroscopic shear modulus as its ideal counterpart. The effective shear modulus is defined as an integral over the thickness of the unit cell, namely:

(7) $$\mu_{eff}=\frac{1}{t}\int_{0}^{t}\mu \left(x\right)dx.$$

Thus, under the uniaxial compression along the layer’s directions, the incompressible hyperelastic layered composite showed the same relation between applied force and displacement as the homogeneous material with shear modulus equal to $\mu_{eff}$. Since the response of the ideal composite and non-ideal composite with linear distribution B were the same for the uniaxial deformation, the observed increase in the stability of the non-ideal composite could not be explained by the change of the effective properties. Therefore, the observed increase in the stability in the non-ideal composite was directly caused by the existence of the smooth transition between soft matrix and stiff layer instead of “jump” or discontinuity of the shear modulus value on the boundary between main homogenous materials. Interestingly, for distribution C, the critical strain remainednearly the same for different values of f, and it may even be lower than the critical strain in ideal layered composite. Similar to case B, in the composite with distribution C, the smooth transition of shear modulus between soft matrix and stiff layer had a stabilizing effect on the uniaxial deformation. However, the effective shear modulus of distribution C was larger than that of the ideal layered composite, which reduced the stabilizing effect of the smooth transition zone. This is reflected by the variation of critical strain for distribution C (see Figure 5).

**Figure 6:**
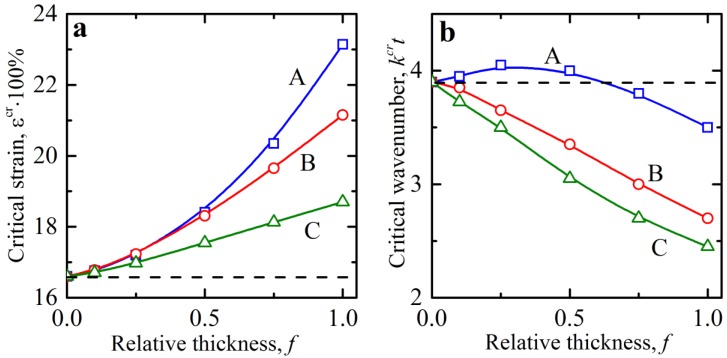
Critical strains (**a**) and wavenumbers (**b**) in the dilute composites with interphasial layers. The volume fraction is $v_{l}=0.025$. Horizontal dashed black line corresponds to the case of ideal composites.

The dependence of critical strain on relative interface thickness f was rather similar for the dilute composite, which underwent buckling by microscopic mechanisms, as shown in Figure 6a. Similar to the non-dilute case, the critical strain increased with an increase in f for shear modulus distributions A and B. At the same time, we did not observe the qualitative difference for dilute composites between the distributions A and C as in the case of non-dilute composite. Non-ideal dilute composites demonstrated more stable behavior (require higher compressive strains for onset of instability) in comparison with their ideal counterparts regardless of the shear modulus distribution in the interphasial layer.

While the non-dilute composites lost their stability by the developing long-wave mode (wavenumber $k^{cr}\to 0$), the dilute composites form wrinkling patterns with finite wavelengths l=1/k. Figure 6b shows the dependence of the critical wavenumber $k^{cr}$ on the relative thickness of the interphasial layer f. We can see that for the shear modulus distributions B and C, the wavenumber drastically decreased with an increase in the interphase layer thickness f. Interesting behavior was observed in the composite with type A shear modulus variation through the interphasial layers. In this case, the dependence of the wavenumber on the relative thickness of the interphasial layer f was not monotonic. In particular, the normalized critical wavenumber increased first, but then started decreasing with a further increase in the interphase thickness, *f*. Note that the effective shear modulus of the combined stiff phase (interphasial zone and stiff layers) for distribution A was lower than the one of the ideal composites; the total effective volume fraction for the combined stiffer zone was increased. However, the smooth change in the local shear modulus—in contrast to the sharp jump in the ideal composite—created a competing stabilizing effect. The contribution of these effects is reflected in the non-monotonic dependence of the normalized wave number for composites with distribution A. For the non-ideal composites with distribution A with higher values of relative thickness of interphasial layer (f≳0.6), buckling modes are characterized by lower normalized critical wavenumber as compared to ideal composites. Gao and Li [34] reported that the layered composites, in which the interphasial layers have a constant shear modulus, can buckle with a formation of patterns where the interphasial and stiff layer have different wavelengths in the buckled mode. These hierarchical patterns appear for specific combinations of geometrical parameters and materials constants [34]. However, in our study—when the transition between two main homogeneous (stiff and soft) constituents was smooth—such hierarchical buckling modes were not observed, and the interphasial and stiff layers demonstrated similar buckling shapes.

Thus far, we considered the ideal and non-ideal composites with the fixed volume fraction of the stiff layer; we examined the interplay between microscopic and macroscopic instabilities and focused on the dependence of critical strain on the volume fraction. From previous studies [3,4], it is known that layered composite undergoes the microscopic type of instabilities if the volume fraction of the stiff layers does not exceed some critical value, depending on the shear modulus contrast. Otherwise, the macroscopic loss of the stability is observed. In the case of the layered composites, containing interphasial layers, we fixed the thickness of the interphasial layer $t_{i}$ and found critical stretch ratio $\lambda^{cr}=1-\varepsilon^{cr}$, for which the composite lost its stability. 

**Figure 7:**
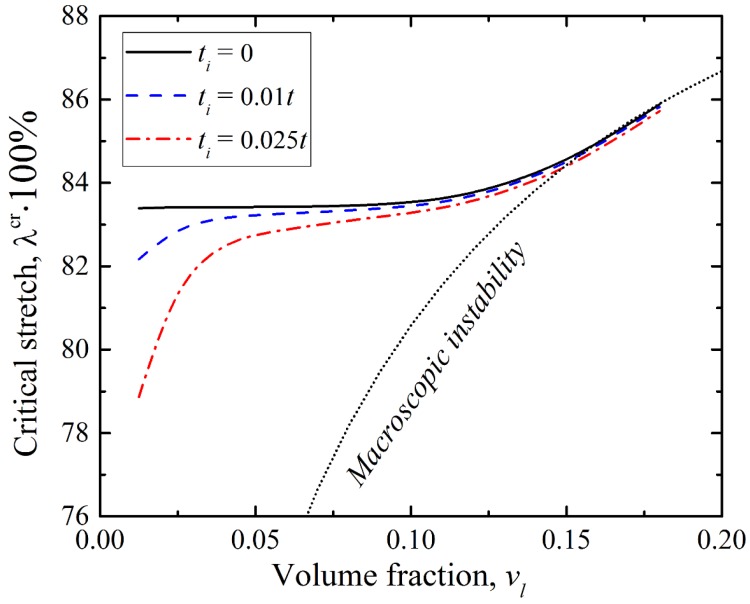
Dependence of the critical stretch ratio $\lambda^{cr}$ on the volume fraction in the layered composite with $\mu_{l}/\mu_{m}=15$ and shear modulus distribution B. The black dotted curve represents the onset of macroscopic instability in the ideal composites (8).

Figure 7 shows the dependence of the critical stretch ratio on the volume fraction for the ideal composite (black continuous curve) and composites with interphasial layers (dashed and dashed dotted curves). We considered the composites with $\mu_{l}/\mu_{m}=15$ and thicknesses of interphasial layers $t_{i}=0,\;0.01t,\;0.025t$. The interphasial layer in these composites had the distribution of the shear modulus, corresponding to the mode B (Figure 2); therefore, all considered composites have the equal effective shear modulus $\mu_{eff}$. The black dotted line represents the onset of the macroscopic instability in the ideal composite, calculated according to the explicit formula [3]:

(8) $$\lambda^{macr}={\left(1-\frac{\hat{\mu }}{\bar{\mu }}\right)}^{\frac{1}{4}\;}$$

where:

(9) $$\bar{\mu }=v_{l}\mu_{l}+\left(1-v_{l}\right)\mu_{m},\;\;\;\;\;\;\;\hat{\mu }={\left(\frac{v_{l}}{\mu_{l}}+\frac{1-v_{l}}{\mu_{m}}\right)}^{-1}$$

From Figure 7, it is clear that the interphasial layers had significant influence on the buckling behavior only if their dimensions were comparable with the dimensions of the stiff layer. In this case, non-ideal layered composite containing interphasial layer were more stable in comparison with ideal composites with the same effective shear modulus. The effect of the interphasial layer decreased with an increase in the volume fraction of the stiff layer, until the transition from microscopic to macroscopic type of instability occurred. At the same time, it should be noted that the observed convergence of the microscopic curves with an increase in volume fraction does not imply that interphasial layers in general have a marginal influence in the composites undergoing macroscopic instabilities. In fact, that Figure 7 shows the results for the composites with fixed $t_{i}$ and not f and, as it was shown in Figure 5b, the interphasial layers played a significant role in the development of instabilities in the non-dilute composites as well.

## 4. Conclusions

The numerical study presented in this paper showed that the existence of inhomogeneous interphases between two main constituents in hyperelastic layered composites significantly changed their buckling behavior, affecting the onsets of the instabilities as well as the developing buckling patterns. In particular, we found that for non-dilute as well as dilute cases, the “non-ideal” composites—which contain the mixing zone between two materials—were usually more stable in comparison with their “ideal” counterparts with the same effective shear modulus. However, it appears that the buckling characteristics depended not only on the thickness and effective shear modulus of the interphasial layer, but also on the distribution of the shear modulus through its thickness. Buckling responses predicted by our analysis were based on certain scenarios for distributions of the properties in the interphase material created due to mixing of the two phases during layer-by-layer curing. A careful experimental characterization is needed to provide the information on these actual distributions, and their dependence on various material fabrication and curing conditions. In the future, it is planned to perform a systematic experimental study of the formed wavy patterns in 3D-printed laminates with controllable interphase mixing zones. In Appendix A, we included a qualitative comparison of the results with limited experimental observations to illustrate the possible effect of the interphase properties on the buckling and postbuckling behavior of the periodic laminates a further careful experimental study should be performed in the future.

## Figures and Tables

**Figure A1:**
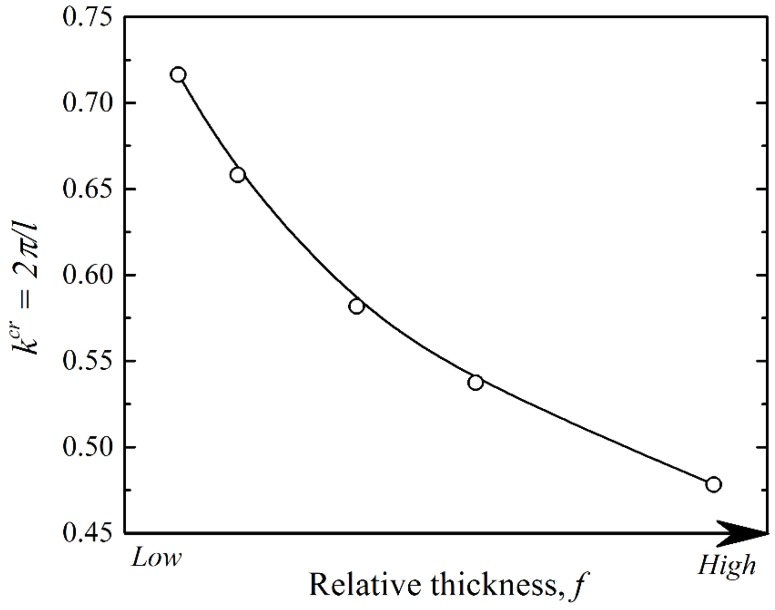
Dependence of critical wavenumber on relative thickness of interphasial layer.

## Appendix A

### Appendix A.1. Experimental Illustration of the Postbuckling Wavy Patterns in 3D Printed Laminates

The dependence of the buckling mode on the relative thickness of interphasial layer, shown in Figure 6a, was used to make some qualitative predictions and estimations of the role of the interphases in 3D-printed layered materials. By using Objet Connex multi-material 3D-printer, we produced the samples, which contain stiff layer embedded in soft matrix. The matrix was printed using soft hyperelastic TangoPlus resin; the layer was printed using so-called digital material DM85 with shear modulus an order of magnitude higher than in TangoPlus [28]. The digital materials in the applied inkjet technology were produced by local mixing of two base materials: TangoPlus and VeroWhite with different mass ratios. During the printing process, the print-head moves above the already printed part of the specimen injecting TangoPlus and VeroWhite inks, and the UV-light cures the deposited layer after each pass. During the inkjet printing, local mixing on the boundary between soft matrix and stiff layer occurs. As a result, the printed composites contain the “pure” homogenous soft matrix (TangoPlus), “pure” stiff homogenous layer (DM85) and the inhomogeneous interphasial layer, where the mixing occurs. We assumed that the amount of TangoPlus in the interphasial layer decreased through the thickness of the interface in the direction towards the stiff homogenous layer. Since the interphasial layer locally is the mixture of TangoPlus and VeroWhite, we treated it as a new “digital” material; and under the assumption that the amount of TangoPlus in the mixed phase decreased towards the stiff layer, we further assumed that the shear modulus of the interphasial layer monotonically increased towards the stiff layer. Another assumption was that the thickness of the interphasial layer or phase mixing zone did not change with the thickness of the stiff layer. Thus, the ratio between thicknesses of interphasial and stiff layers f changed with the since the thickness of the stiff layer. In accordance to the numerical results, shown on Figure 6a, we performed compression experiments on the 3D-printed composites with different thicknesses of stiff layer in order to measure the wavelengths of the buckling patterns. We used only one layer in our experiments; however, the critical strain and wavelength in this case matched the critical strain and wavelength of the corresponding dilute composite almost perfectly.

Table A1 shows the experimental snapshots for the composites with different thicknesses of the embedded stiff layer. Regardless of the layer thickness, we observed that buckling occurred in the microscopic scenario with the formation of wrinkling patterns. Under the assumptions above about the interphase and the measurements of the wavelengths, estimates for the dependence of critical wavenumber $k^{cr}$ on the relative interface thickness can be summarized as shown in Figure A1. We did not know exactly the thickness of the interphasial layer, and we could only qualitative follow the change in k with an increase in the relative thickness of the interphasial layer f.

**Table A1:** Observed buckling patterns for the samples with different thickness of the stiff layer $t_{l}$.

$t_{l}$ (mm)	Observed Buckling Pattern
0.75	
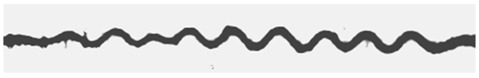
1.0	
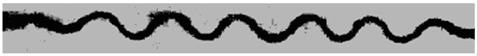
1.5	
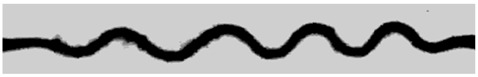
2.0	
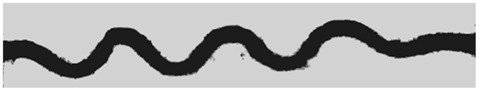

As one may see from Figure A1, wavenumber $k^{cr}$ decreased with an increase in the relative thickness f. A qualitative comparison of the experimental observations with the numerical results (see Figure 6b) may hint that the interphasial layers in the studied 3D-printed composites has the distribution of the shear modulus through the thickness similar to the cases B or C (Figure 2). The mixing mass ratio between TangoPlus and VeroWhite in the interphasial layer might change linearly through the thickness of the interphasial layer; however, the change in the shear modulus through the thickness of the mixing zone might be highly non-linear. This may explain the observed dependence of $k^{cr}$ on relative thickness f. Thus, the combination of the experimental observations and numerical simulations potentially may be used for indirect characterization of interphasial layers in 3D-printed soft composites, based on their buckling behavior.
